# A 68-year-old Caucasian man presenting with urinary bladder lymphoepithelioma: a case report

**DOI:** 10.1186/1752-1947-7-161

**Published:** 2013-06-20

**Authors:** Gian Paolo Spinelli, Giuseppe Lo Russo, Alberto Pacchiarotti, Valeria Stati, Alessandra Anna Prete, Federica Tomao, Cinzia Sciarretta, Mara Arduin, Enrico Basso, Stefania Chiotti, Marsela Sinjari, Martina Venezia, Giada Zoccoli, Silverio Tomao

**Affiliations:** 1 UOC Oncology Aprilia - (LT), University of Rome ‘Sapienza’, via Giustiniano snc, 04011 Aprilia, LT, Italy; 2 Division of Anatomo-Pathology- Aprilia, via Palme, 25, 04011 Aprilia LT Italy; 3 Department of Gynaecology and Obstetrics, University of Rome ‘Sapienza’, viale Regina Elena 324, 00161 Rome, Italy; 4 Department of Medical-Surgical Science and Biothecnology, University of Rome ‘Sapienza’, Corso della Repubblica 04100, LT, Italy

**Keywords:** Bacillus Calmette-Guérin Instillations, Lymphoepithelioma-like Carcinoma, Urinary Bladder

## Abstract

**Introduction:**

Lymphoepithelioma is a very rare form of malignant tumor originating from epithelial line cells. Its occurrence has potential clinical, therapeutic and prognostic implications. In the present report we describe an unusual case of bladder cancer with two different histological varieties: transition cell carcinoma and lymphoepithelioma-like carcinoma. Lymphoepithelioma-like carcinoma of the bladder has only been rarely reported in the literature to date.

**Case presentation:**

We present the case of a 68-year-old Caucasian man who, after occurrence of hematuria, underwent transurethral resection of a bladder tumor. The results of a histological examination confirmed a high-grade non-muscle-invasive pT1 lymphoepithelioma-like carcinoma of the urinary bladder, associated with a concurrent high-grade transition cell carcinoma. After analyzing the histological features, our patient was subjected to treatment with intra-vesical instillations of bacillus Calmette-Guérin. Our work stresses that diagnosis and therapeutic approaches can be difficult and controversial, especially in the early stages of this rare carcinoma.

**Conclusions:**

This report emphasizes the importance of extending our knowledge and experiences regarding this uncommon carcinoma. Further studies are needed to better understand this rare disease and define more accurate diagnostic and therapeutic strategies.

## Introduction

Lymphoepithelioma-like carcinoma (LELC) is a rare form of poorly differentiated or undifferentiated malignant tumor originating from epithelial line cells, described for the first time in the nasopharynx [1].This unusual form of carcinoma has subsequently been described in several tissues, including the salivary glands, soft palate, thymus, trachea, lung, cervix, skin, stomach, breast, and whole urinary tract [2]. The most frequent site in which it can develop seems to be head and neck region, while the urinary tract is an extremely rare localization. Among LELCs arising in the urinary tract, the bladder represents the most common site. Since 1991 no more than 80 cases of urinary bladder LELC have been described [2,3] and it is estimated that this kind of carcinoma represents 0.4 to 1.3 percent of all bladder cancers [4].

The histological features of LELC include an inflammatory infiltrate and conspicuous lymphoid aggregates; furthermore, syncytia of cells and prominent nucleoli can be observed. The number of mitoses is usually high. Regardless, the pathognomonic finding is the presence of both T and B lymphocytic infiltrate; however, other inflammatory cells have also been described such as eosinophils and plasma cells. Immunochemical test results are also important in order to demonstrate the presence of cytokeratins; in fact, these prove the epithelial origin of the tumor [5]. Recognizing this rare histological form of carcinoma is important not only for diagnosis, but also for clinical, therapeutic and prognostic implications. The present article describes an interesting case of bladder cancer in which two different histological varieties were observed: transition cell carcinoma (TCC) and LELC.

## Case presentation

**Figure 1:**
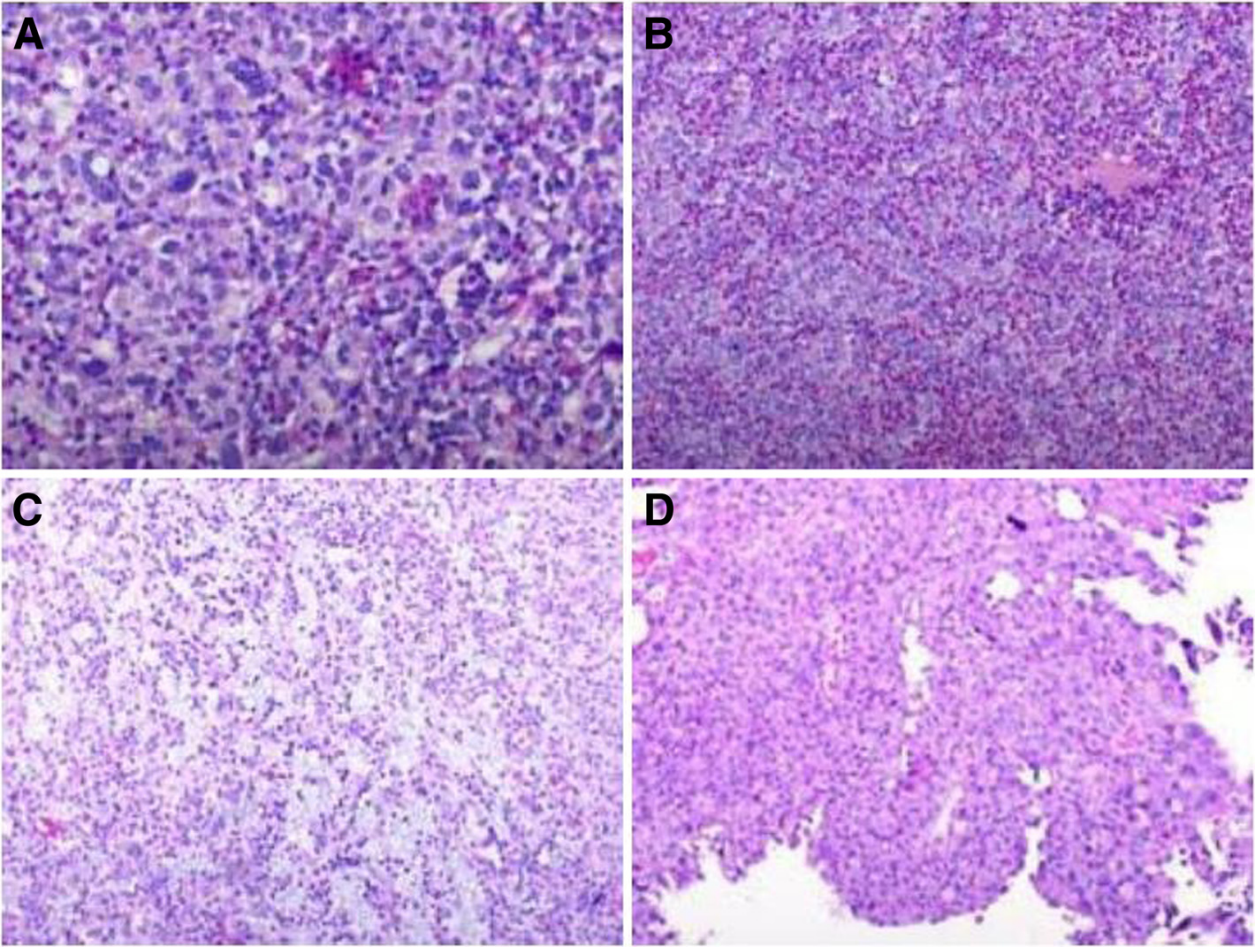
Histologically, lymphoepithelioma is composed of nests, sheets and cords of undifferentiated cells with large pleomorphic nuclei and prominent nucleoli, and the cytoplasm borders are poorly defined imparting a syncytial appearance (A,B); the background consists of a conspicuous lymphoid stroma with histiocytes, neutrophils and eosinophils, the latter prominent in our patient’s case (C); the tumor is focally admixed with typical high-grade papillary urothelial carcinoma (D).

**Figure 2:**
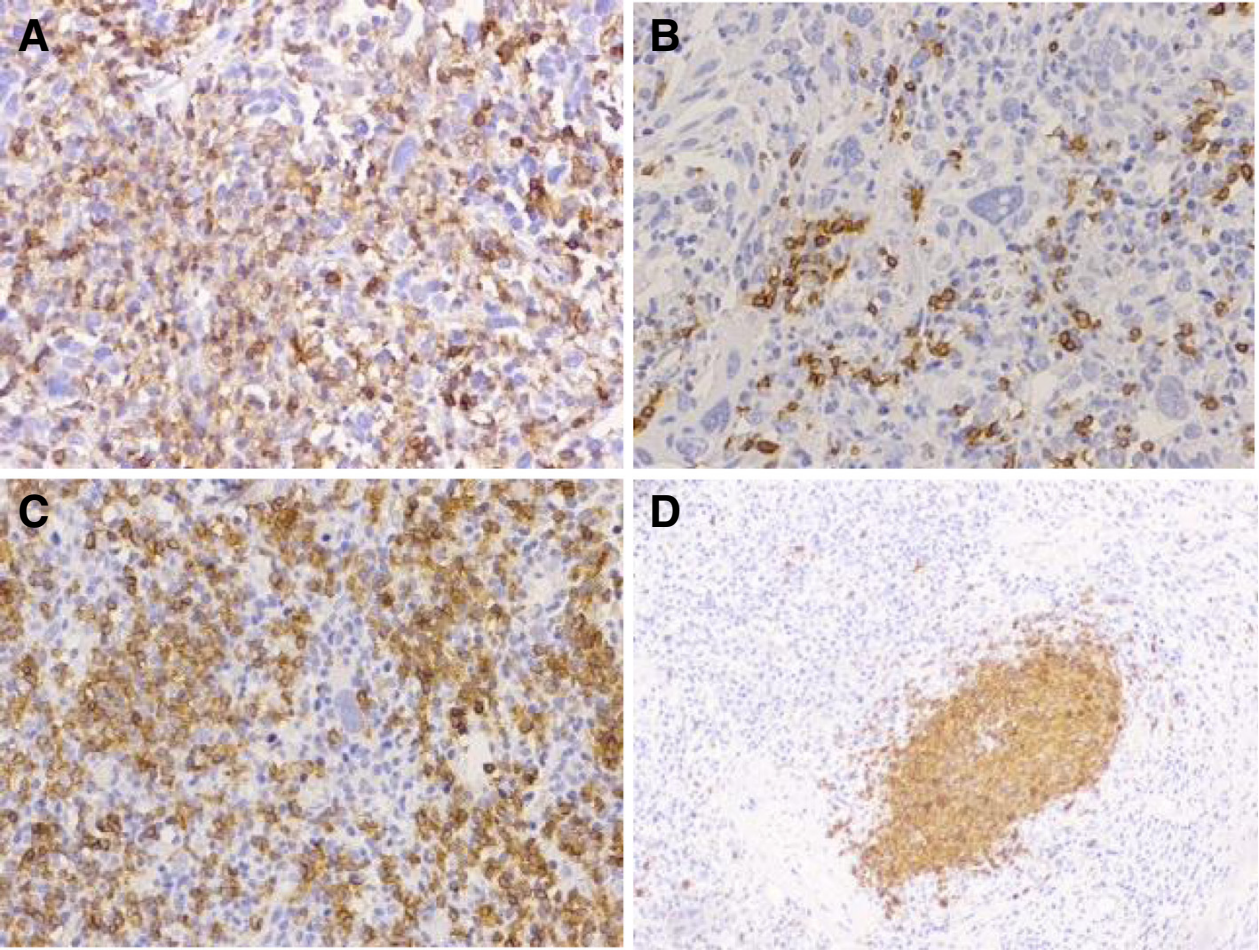
**High-grade non-muscle-invasive lymphoepithelioma-like carcinoma of the urinary bladder associated with a concurrent high-grade transitional cell carcinoma.** Lymphoid stroma, immunostained with CD45/pan-Leu (**A**), includes plasma cells, CD3-positive T lymphocytes (**B**) and CD20-positive B lymphocytes, the latter diffuse (**C**) and in follicles (**D**).

A 68-year-old Caucasian man presented to our facility with a three-week history of hematuria. Our patient was a non-smoker and had not been exposed to carcinogenic material during his lifetime. He had no history of urological problems. On an ultrasonography scan of his abdomen and pelvis, a 28×13mm hyper-echoic lesion on the right side wall projecting into the bladder was observed. No other lesions were noted on the bladder, ureter, or renal pelvis. A transurethral resection of bladder tumor (TURBT) was performed, and the subsequent histological examination revealed a predominant inflammatory infiltrate with lymphoid aggregates and a high number of mitoses. The histological diagnosis was high-grade non-muscle-invasive pT1 LELC of the urinary bladder associated with a concurrent high-grade TCC (Figures 1 and 2). Consequently, a total body computed tomography (CT) scan was performed and the presence of distant metastases or lymph node involvement was excluded. After discussion with our patient and his family, treatment with intra-vesical instillations of bacillus Calmette-Guérin (BCG) strain TICE® containing 200 million colony-forming units of BCG was carried out as prophylaxis to prevent bladder cancer recurrences. The schedule comprised a weekly instillation for the first six weeks, followed by monthly maintenance BCG therapy for a period of 12 months. The immunotherapy was well tolerated by our patient. No grade 3 toxicities were reported, and the only adverse events were strangury and microscopic hematuria. During instillation therapy, our patient’s urine cytology showed some atypical cells. Therefore, cystoscopy was performed but this did not show any remaining malignancy. A urological evaluation was obtained and another TURBT was scheduled after performing Uro-computed tomography (Uro-CT). At the end of the treatment, a Uro-CT scan was carried out and the results were negative. Therefore, our patient did not undergo a second TURBT procedure. Despite this result, multiple bladder biopsies were performed and excluded LELC recurrence. Subsequently our patient has been included in a program of periodic follow-up examinations. At present, he is free from disease.

## Discussion

LELC is an extremely rare variant of carcinoma, first described in the bladder by Zuckerberg *et al.*[4]. Since 1991 there have been only a few relatively small studies on LELC of the urinary bladder [3,6-9]. The exact pathogenesis of this tumor is not well established. Epstein-Barr virus is frequently associated with lymphoepithelioma of the nasopharynx and other tissues (lung, stomach, thymus and salivary gland). However, this association has not been documented for LELC of the urinary bladder [10]. It has been suggested that abnormalities of p53 regulation might be crucial in the pathogenesis of LELC of the urinary bladder [8]. The exact origin of this carcinoma is not well known, but the expression of common urothelial markers suggest that it could arise from stem cells [9]. LELC of the urinary bladder must be distinguished from lymphoma or reactive inflammatory lesions. In our patient’s case the differential diagnosis of bladder LELC from lymphoma (very rare bladder carcinomas) was undertaken with the help of immunohistochemistry test results for cytokeratin and the lymphoid markers CD45/pan-Leu, CD3 and CD20. Given the presence of an inflammatory background, neoplastic cells may be exchanged for reactive histiocytes and the lesion may be confused with chronic cystitis. Therefore, immunohistochemistry should also be used to exclude a histiocytic nature and avoid a misdiagnosis of chronic cystitis [4]. Another important issue is to distinguish LELC associated with poorly differentiated invasive TCC from poorly differentiated squamous cell carcinoma with associated dense lymphoplasmacytic infiltrate [11]. Uncommonly, LELC may be accompanied by adenocarcinoma and squamous cell carcinoma as well, and some reports have described this association [11]. LELC may also develop as a pure form or more often in combination with other kinds of neoplasm, such as TCC. Much evidence suggests that TCC generally has a worse prognosis than LELC, while focal LELC is expected to be more aggressive than the pure form [6,7]. It is likely these changes in prognosis are associated with the immune response generated by these lymphoid cells against the tumor. Amin *et al.* created a classification to distinguish among various possibilities of association. Neoplasms were categorized according to the proportional expression of lymphoepithelial elements in the tumor mass as pure (all lymphoepithelial elements), predominant (more than half) or focal (less than half) [1]. In the present work, we describe a rare case of predominant LELC associated with a concurrent high-grade TCC. Our patient was alerted by early symptoms (macroscopic hematuria and dysuria) that several investigators have suggested are caused promptly by the inflammatory infiltration that characterized this carcinoma [9].

The prognosis of pT1 LELC is generally positive, but gets progressively worse with increasing stage (pT2-4).

As a result of the low incidence of this carcinoma, no standard approach exists. There are several alternatives, depending on the tumor stage, which include TURBT and intra-vescical instillations in pT1 bladder LELC, and cystectomy with or without chemotherapy or radiotherapy for advanced stage disease [2,6,7].

It is also known that pure/predominant LELC may respond to chemotherapy and may be treated with bladder preservation. Unfortunately, the very low incidence of this type of neoplasm and the limited experience in therapeutic approaches to bladder LELC makes comparison among these different subtypes difficult.

Tamas *et al.* conducted a study on 30 cases of LELC, at various stages and with different histology types (pure, predominant and focal). A total of 13 of these patients (two with pT1 tumor, four with pT2, five with pT3 and two with pT4) underwent cystectomy, with or without prostatectomy. A five-year follow-up demonstrated a recurrence-free risk of about 59 percent, independent of histological subtype (pure, predominant or focal) [2]. In the literature, few reports underline the best treatment in cases of LELC at the pT1 stage. This may be due to the fact that the pT2-T3 stages seem to be the most frequent presentations of LELC at diagnosis [3]. In the Tamas *et al.* study, only two patients at the pT1 stage who were TURBT treated were enrolled. These patients were free from disease at 48 months [2].

In the treatment of LELC, different chemotherapy regimens have been used but the limited number of reported cases makes the comparison difficult. In several studies platinum-based regimens have been seen as reasonable options, especially as primary chemotherapy [12,13], but the evidence suggests that both cystectomy and chemotherapy are indicated only for LELC from stage T2 onwards. Instead, for pT1 LELC authors have suggested intra-vesical instillations with epirubicin, mitomycin or BCG [14]. In our patient, pT1 stage at diagnosis and the presence of a concurrent high-grade TCC component prompted us to treat the tumor with TURBT followed by BCG instillations. Early stage LELC has only rarely been described in the scientific literature; our patient’s case suggests that bladder-preserving therapy may also be used to successfully treat predominant LELC.

## Conclusions

In summary, our experiences with our patient as outlined in this report underline the main problems associated with bladder LELC. First, differential diagnosis can be difficult. Therefore, in our opinion diagnosis should be performed preferably by an experienced pathologist. Second, due to the lack of information regarding the best therapy for each stage and the lack of specific guidelines, treatment is based mainly on the results of a few reviews and case reports. In our patient’s case, a pT1 LELC of the urinary bladder was treated with BCG instillations. Although the follow-up period has not yet been completed, our patient is currently free from disease. Other studies are necessary to better understand this carcinoma, and to define more accurate diagnostic and therapeutic approaches.

## Consent

Written informed consent was obtained from the patient for publication of this case report and any accompanying images. A copy of the written consent is available for review by the Editor-in-Chief of this journal.

## Competing interests

The authors declare that they have no competing interests.

## Authors’ contributions

GPS, GLR, VS, AAP, FT and ST designed the study, analyzed and interpreted the data from our patient and drafted the manuscript. AP performed the histological examination of the bladder. CS, MA, EB, MS, MV, GZ contributed to the work. All authors read and approved the final manuscript.
